# Seminal plasma protects frozen-thawed ram spermatozoa from neutrophil attack: a complement mediated dynamic

**DOI:** 10.1530/REP-24-0465

**Published:** 2025-07-04

**Authors:** Sophie Warr, Taylor Pini, Simon P de Graaf, Jessica P Rickard

**Affiliations:** ^1^ School of Life and Environmental Sciences, Faculty of Science, The University of Sydney, Sydney, New South Wales, Australia; ^2^ School of Veterinary Science, Faculty of Science, The University of Queensland, Gatton, Queensland, Australia

**Keywords:** cryopreservation, sheep, immune, cervical AI

## Abstract

**In brief:**

Frozen-thawed ram spermatozoa show reduced fertility following cervical artificial insemination, likely attributed to an elevated immune response in the ewe. This paper identifies the immunoprotective effect of ram seminal plasma (SP) against polymorphonuclear neutrophil binding, highlighting potential for fertility improvement.

**Abstract:**

Following cervical artificial insemination, frozen-thawed (FT) ram spermatozoa display reduced fertility compared to fresh spermatozoa, irrespective of sperm motility and viability, likely attributed to an elevated immune response in the ovine cervix. SP has previously been shown to be beneficial to sperm transport in the ovine cervix, yet the components responsible for this protective effect remain unknown. As such, the present study examined the immune dynamics of frozen-thawed ram spermatozoa with polymorphonuclear neutrophils (PMNs), utilising a neutrophil binding assay. The effect of 25% v/v SP supplementation on binding susceptibility was also investigated. A portion of SP was heat-treated before incubation (HTSP) to isolate the impact of SP proteins. The presence of SP significantly reduced sperm-PMN binding (37.15 ± 0.02%, 46.59 ± 0.02%, 38.83 ± 0.02%) compared to FT alone (62.83 ± 0.02%, 75.74 ± 0.02%, 56.0 ± 0.03%) across all serum groups (no serum, serum and heat-treated serum, respectively). HTSP showed comparable binding susceptibility to the FT treatments, indicating that the protective effect of SP is attributed to a heat-labile factor. Serum significantly increased sperm-neutrophil binding irrespective of SP treatment. However, this was reduced to serum-free levels following heat-treatment, suggesting sperm-neutrophil dynamics are further mediated by protein components within ewe serum, likely complement proteins. The viability of sperm or the presence of freezing medium did not influence PMN binding. Furthermore, PMN viability and therefore binding were not influenced by the presence of serum or SP. Together, the current study presents new evidence for the immunoprotective properties of SP in sheep, which could be leveraged to aid transit through the cervix.

## Introduction

Successful fertilisation requires the precise alignment of a series of coordinated physiological processes involving both male and female counterparts. Any disruption in timing, biochemical interactions or molecular structures can lead to reproductive failure (Gadella 2009, Purdy *et al.* 2020, Abril-Parreño *et al.* 2022). Artificial reproductive technologies (ARTs) require a comprehensive understanding of the male-female dynamic to achieve pregnancies. In sheep, cervical artificial insemination (AI) with frozen-thawed semen is not widely utilised due to unacceptably low pregnancy rates (typically <30%) (Salamon & Lightfoot 1970, Kaabi *et al.* 2006). The reason for these low pregnancy rates is linked to passage through the cervix, as bypassing the cervix and surgically depositing frozen-thawed semen directly into the uterine horns achieves high pregnancy rates (typically >70%) (Salamon & Maxwell 2000). In comparison to freshly ejaculated sperm, seminal plasma-naïve epididymal spermatozoa also demonstrate poor cervical transit (Maxwell *et al.* 1999, Rickard *et al.* 2014). This suggests that inherent differences in the capacity to successfully fertilise following cervical AI are tied to passage through the ovine cervix and likely linked to the presence of seminal plasma (Maxwell *et al.* 1999, Rickard *et al.* 2014, Warr *et al.* 2023).

To make meaningful progress in improving pregnancy rates following cervical insemination with frozen-thawed ram semen, the causes underlying this failure of cervical progression require further investigation. Decades of work have gone into characterising the physiological identity of ‘fertile’ spermatozoa, identifying parameters that increase the chances of fertilisation (Vijayaraghavan 2003, Gillan *et al.* 2005, Van de Hoek *et al.* 2022). While reduced post-thaw motility of cryopreserved sperm has been correlated to reduced *in vivo* fertility in sheep (Gillan *et al.* 2008, Furstoss *et al.* 2010), it does not fully account for lower pregnancy rates following cervical AI (Salamon & Maxwell 2000, O’meara *et al.* 2005). It is therefore evident that traditional parameters of sperm fertility are not solely responsible for successful cervical transit.

The female reproductive tract presents a host of physical, biochemical and immune barriers to ascending spermatozoa (for review see Warr *et al.* (2023)). We hypothesise that while fresh, ejaculated spermatozoa are properly equipped to overcome these physiological barriers, epididymal and frozen-thawed spermatozoa are unable to overcome these challenges and successfully traverse the female tract. In sheep, semen is deposited in the anterior vagina at the cervical *os*. Thus, the first barrier they encounter in their passage to fertilisation is the cervix. In the ewe, a series of misaligned cartilaginous folds presents a unique challenge, minimising ‘straightforward’ passage towards the uterine body (Halbert *et al.* 1990). Within the cervix, spermatozoa are faced with the biophysically dynamic obstacle of cervical mucus (Katz *et al.* 1997, Kölle 2015). In addition, the introduction of semen induces production of inflammatory cytokines (Sharkey *et al.* 2007, Scott *et al.* 2009), resulting in the rapid influx of immune cells into epithelial tissues (Sharkey *et al.* 2012) and the lumen of the female reproductive tract (Mattner 1969, Robertson *et al.* 1996), occurring within one hour of insemination (Phillips & Mahler 1977, Rozeboom *et al.* 1998, Ribeiro *et al.* 2006). Once in the tract, neutrophils form close associations with sperm either through direct sperm and neutrophil binding or formation of neutrophil extracellular traps (NETs) to initiate phagocytosis (Troedsson *et al.* 2001, Alghamdi *et al.* 2004, Alghamdi *et al.* 2009, England *et al.* 2013). Previous studies by Pini *et al.* (2016) utilised a neutrophil binding assay to observe sperm-neutrophil interactions and found that the presence of heat-treated serum, seminal plasma and cryopreservation significantly alter the interactions of spermatozoa with neutrophils. It was theorised that these differences could potentially underlie differences in cervical transit, with immune-modulatory factors within seminal plasma and changes to sperm surface molecules hypothesised to be involved. The specific sperm surface molecules that mediate neutrophilic interactions remain unclear; however, complement factors have previously been proposed in other livestock species (Taylor *et al.* 2008, Alghamdi *et al.* 2009).

The complement system plays an important role in host defence and inflammation, consisting of a tightly regulated network of proteins. Increased activation of complement has been shown to increase chemotaxis, leukocytic migration and opsonisation of foreign cells (Clark & Klebanoff 1976). Opsonised cells are subsequently removed by phagocytic cells or lysed by membrane attack complex (MAC). Thus, complement-mediated immune responses may be key in neutrophil recognition and interception of sperm cells in the female tract. Complement activation occurs through three separate pathways: alternate, classical and lectin (Sarma & Ward 2011). The alternate pathway is triggered by carbohydrates, lipids and proteins, which are found on ‘non-self’ surfaces. C3, found in serum, is constantly hydrolysed at a low level to form C3b, which binds the factor B product to create the C3 convertase C3bBb. The classical pathway is initiated when immune complexes are formed following IgG/IgM binding to pathogens or non-self antigens. Following activation, C1 binds the exposed Fc portion of IgG/IgM and subsequently cleaves C4 and C4b to form the C3 convertase C4bC2a. The lectin pathway is activated when either mannose-binding lectin or ficolin binds to carbohydrate moieties on the surfaces of pathogens and shares C3 convertase C4bC2a formation with the classical pathway. Human and bovine spermatozoa have been suggested to activate complement via the alternate pathway, having no neutrophil chemotactic activity alone but potent activity in the presence of serum (Clark & Klebanoff 1976). One outcome of an elevated response is increased neutrophil activity and elimination of opsonised cells. Seminal plasma has been shown to influence PMN binding to epididymal ram sperm (Pini *et al.* 2016); however, its protective effect on frozen-thawed ram spermatozoa is yet to be fully elucidated. Future research is needed to further investigate factors within seminal plasma that may influence sperm-PMN interactions. The role of serum also needs to be studied when investigating the potential anti-complement activity of seminal plasma described in other species (Brooks *et al.* 1981, Price *et al.* 1984). The inclusion of serum, which introduces factors such as immunoglobulins and complement proteins, enables activation of complement-mediated immune responses such as opsonisation of cells. Heat-inactivation of serum allows the effect of complement proteins to be excluded. In a serum-free environment, the role of non-opsonin binding (e.g. via selectins, lectins or integrins) can be assessed. By investigating the factors driving and limiting sperm-neutrophil interaction, we seek to identify targets for intervention to suppress the female immune response to frozen-thawed spermatozoa.

The present study aims to utilise a neutrophil binding assay to determine the effect of cryopreservation and seminal plasma on sperm-neutrophil interactions, with a focus on distinguishing between immune pathways that might be responsible for mediating these interactions. We hypothesise that changes to the molecular profile of sperm during cryopreservation will alter sperm-neutrophil interactions. Furthermore, the role of seminal plasma and its heat-labile factors in sperm-PMN binding will be investigated via supplementation of seminal plasma and heat-treated seminal plasma. We also aim to demonstrate the role of complement in this dynamic through the modulation of available complement factors through the inclusion of serum treatments.

## Materials and methods

### Experimental design

Four studies were designed to investigate changes in sperm-neutrophil dynamics following cryopreservation and supplementation with seminal plasma. Please refer to Supplementary Table S1 (see section on Supplementary materials given at the end of the article) for a representation of the treatment design for all studies contained within.

The first study (*n* = 3 rams × 3 ejaculates) used a neutrophil binding assay (NBA) to compare the ability of fresh ejaculated spermatozoa to bind to PMN compared to frozen-thawed ejaculated spermatozoa. To further examine mechanisms underpinning these interactions, sperm treatments were incubated in the presence or absence of intact and heat-inactivated ewe serum (7.5% v/v).

In study two, the influence of sperm viability and diluent on neutrophil binding affinity was investigated, comparing PMN binding in a ‘non-viable’ versus ‘viable’ sperm treatment and egg-yolk supplemented (CRYO) vs non-egg-yolk supplemented extender (SSF; *n* = 3 rams × 4 ejaculates).

To investigate how seminal plasma may influence immune-based interactions, the third study (*n* = 3 rams × 4 ejaculates) compared PMN binding in frozen-thawed spermatozoa (FT), frozen-thawed spermatozoa supplemented with 25% seminal plasma (FTSP) and 25% heat-inactivated seminal plasma (FTHSP), again in the absence of serum, with serum or with heat-treated serum (7.5% v/v). Here, the concentration of terminal complement factor C5 was also assessed following incubation of sperm with PMNs, via an enzyme-linked immunosorbent assay (ELISA), with isolated whole seminal plasma as a control.

Finally, in study 4, the effect of seminal plasma and serum on PMN viability was assessed. Isolated PMNs (*n* = 2 ewes × 2 technical replicates) were incubated for 120 min at 37°C with ewe serum (7.5% v/v) and seminal plasma (25% v/v). Aliquots were taken at 0, 30 and 120 min for staining with Trypan blue, and the number of stained (non-viable) vs non-stained (viable) PMNs counted and presented as a percentage.

### Chemicals

All chemicals were purchased from Sigma-Aldrich (Australia) unless otherwise stated. Ewe serum (S3772) was purchased from Sigma and heat-treated at 56°C for 30 min to inactivate complement, then frozen in aliquots and stored at −20°C until use.

### Animal use and ethical approval

Mature Merino rams (*n* = 3) used for semen collection and Merino ewes (*n* = 2) used for blood collection were kept on a chaff-based diet supplemented with lupins in an animal house at the University of Sydney, Camperdown, NSW, Australia. All work was approved by the University of Sydney Animal Ethics Committee (Project No.: 2023/2277).

### Seminal plasma collection and treatment

Seminal plasma was obtained from ejaculates (*n* = 6) collected from mature Merino rams (*n* = 3) in the presence of a teaser ewe. Semen was collected via artificial vagina and assessed immediately for quality by scoring wave motion (0–5). Samples with a wave motion <3 were excluded. Semen was then centrifuged at 12,000 ***g***, 4°C for 10 min, the supernatant aspirated and spun for a further 10 min at 12,000 ***g***, 4°C to remove any remaining spermatozoa and cell debris. Seminal plasma samples were then separated into aliquots and stored at −80°C. On the day of an experiment, seminal plasma was thawed on ice, ejaculates pooled by ram, and a portion was heat-inactivated in a water bath for 45 min at 90°C (Troedsson *et al.* 2005).

### Processing and assessment of sperm treatments

Semen was collected via artificial vagina in the presence of a teaser ewe and assessed immediately for quality by scoring wave motion, concentration and volume. Samples with a wave motion <3 were excluded. Fresh samples were diluted to 400 × 10^6^ spermatozoa/mL in Salamon’s cryodiluent (CRYO; 300 mM Tris, 28 mM glucose, 104 mM citric acid, 15% (v/v) egg yolk, 5% (v/v) glycerol, pH 7.3, mOsm 295±5) (Evans & Maxwell 1987). Frozen-thawed samples were diluted to 400 × 10^6^ spermatozoa/mL in Salamon’s cryodiluent, chilled to 4°C over 2 h, then frozen on dry ice in pellets of 250 μL and stored in liquid nitrogen (Evans & Maxwell 1987). Pellets were thawed in a 37°C water bath with agitation for 2 min. Fresh and frozen-thawed spermatozoa were then washed using a Percoll gradient (4 mL 35%, 2 mL 70%; 5 min 200 ***g***, 15 min 1,000 ***g***) and the subsequent pellets washed (10 min 800 ***g***) in Tris-citrate-fructose (SSF; 300 mM Tris, 94.7 mM citric acid, 27.8 mM fructose, 1% v/v penicillin and streptomycin) (Evans & Maxwell 1987). Both fresh and frozen-thawed treatments were then brought to 50 × 10^6^ spermatozoa/mL in SSF supplemented with bovine albumin serum (SSF + BSA; 0.3% v/v BSA) before incubation with PMNs.

In study 2, sperm viability and diluent type were assessed. Before PMN addition, an aliquot of fresh ejaculated spermatozoa was treated by repeated freeze–thaw in liquid nitrogen, which served as a non-viable control to investigate the contribution of viability to neutrophil binding susceptibility. Fresh ejaculated spermatozoa were prepared as above and diluted to 50 × 10^6^ spermatozoa/mL in CRYO or SSF before incubation with PMNs.

In study 3, frozen-thawed samples were washed as described above and diluted to 100 × 10^6^ spermatozoa/mL in SSF + 0.3% BSA. A 1:1 (v/v) dilution was then performed with either SSF + 0.3% BSA (FT), seminal plasma (50% v/v FTSP) or heat-treated seminal plasma (50% v/v FTHTSP) with a final sperm concentration of 50 × 10^6^ spermatozoa/mL.

### Sperm quality assessment

Immediately before incubation with PMNs, sperm motility was measured using computer-assisted sperm analysis (HT CASA IVOS II (Animal Breeder) Version 1.13.7; Hamilton-Thorne, USA) using the appropriate settings for ram spermatozoa (this includes, among others; head size 10–42 μm2, progressive motility thresholds of straightness 80% and average path velocity 75 μm/s). Samples were further diluted to a concentration of 25 × 10^6^ sperm/mL with PBS + 0.3% BSA before 6 μL was placed on slides warmed to 37°C (Cell Vu; Millennium Sciences, Australia) and enclosed with a 22 × 22 mm coverslip. For each sample, eight fields of video recordings were recorded, capturing a minimum of 200 cells (frame rate 60 Hz). Motility and kinematic parameters were subsequently calculated including total and progressive motility.

Samples were assessed for viability and acrosome integrity at a final concentration of 10 × 10^6^ sperm/mL using the CytoFLEX flow cytometer (CytExport 2.0 Software, Beckman Coulter; USA), with three lasers: 50 mW 488 nm, 50 nW 638 nm and 80 mW 405 nm. All samples were stained with the DNA probe Hoechst 33,342 (final concentration 1 μg/mL), detected via a 450/45 BP filter, to gate any possible debris in the sample. Sample preparation for sperm viability and acrosome integrity was performed as previously described (Pool *et al.* 2020), by staining with a combination of propidium iodide (PI, final concentration 6 μM) and fluorescein isothiocyanate peanut agglutinin (FITC-PNA, final concentration 0.4 μg/mL) for 10 min at 37°C. PI and FITC-PNA fluorescence detection was on 690/50 and 525/40 nm BP filters respectively. Cells were considered viable with intact acrosomes if cells were both PI and FITC-PNA negative.

### Isolation of polymorphonuclear lymphocytes

Blood was collected from the jugular vein of mature Merino ewes (*n* = 2) into ethylenediaminetetraacetic acid (EDTA)-coated vacutainers (Becton Dickinson, Australia) and pooled. PMNs were isolated as per the protocol outlined by (Pini *et al.* 2018*b*) immediately following collection. Briefly, blood was layered onto a two-phase gradient of Histopaque-1119 and Histopaque-1077 and centrifuged (3 mL Histopaque-1119, 3 mL Histopaque-1077, 6 mL whole blood, 1,200 *g*, 30 min, room temperature). The PMN layer was recovered and red blood cells removed by 30 s hypotonic lysis with ultrapure water. PMNs were washed twice in phosphate-buffered saline (PBS; 137 mM NaCl, 3 mM KCl, 8 mM Na2HPO4, 1 mM KH2PO4). Cell viability was determined by Trypan blue exclusion as previously described by (Pini *et al.* 2018*b*) and was consistently high (>95%). Immediately before mixing with spermatozoa, PMNs were resuspended to 5 × 10^6^ PMN/mL with PBS + 15% (v/v) ewe serum (S) and heat-inactivated (H-S) ewe serum for studies one and three.

### Sperm-PMN binding assay

A sperm-PMN assay was conducted as reported by (Pini *et al.* 2018*b*). Spermatozoa treatments at a concentration of 50 × 10^6^ spermatozoa/mL were further diluted 1:1 (v/v) with isolated PMNs (5 × 10^6^ PMN/mL) in PBS (no serum), serum (15% v/v S) or heat-inactivated serum (15% v/v H-S), resulting in final concentrations of 25 × 10^6^ spermatozoa/mL, 2.5 × 10^6^ PMN/mL and 7.5% (v/v) serum, 25% seminal plasma respectively. Samples were then incubated in a dry bath incubator (Major Science, USA) at 37°C. After 30 and 120 min, samples were mixed vigorously with a pipette to dissociate large cell clumps, and a 10 μL aliquot was smeared on a labelled microscope slide and air-dried thoroughly.

**Figure 1:**
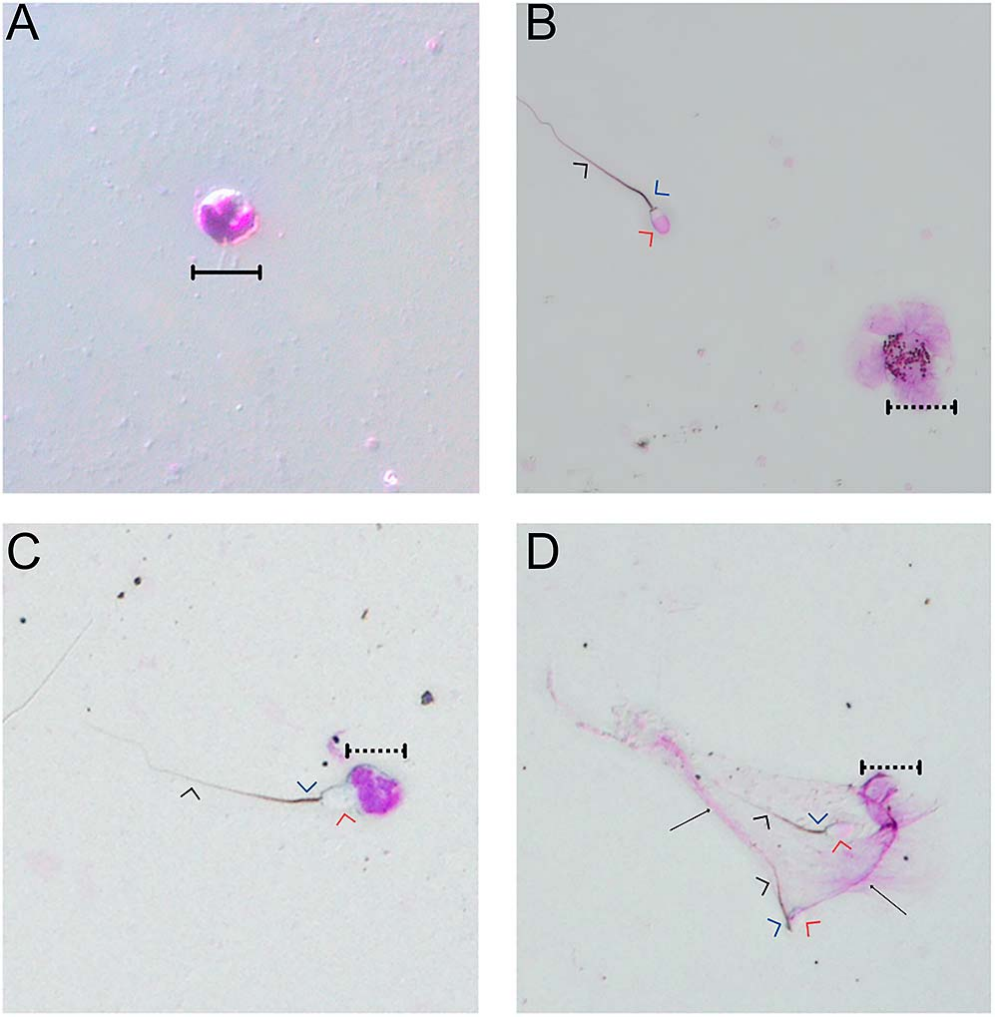
DIC images of ram spermatozoa interactions with polymorphonuclear neutrophils (PMNs) at 40× magnification following Wright’s staining. Arrowheads indicate the ram spermatozoon head (red), midpiece (blue) and tail (black). Solid brackets indicate inactive PMNs while dashed brackets indicate activated PMNs. NETs are denoted by a solid black arrow. (A) Inactive PMN not bound to spermatozoa, characterised by a defined multi-lobular nucleus and intact plasma membrane.(B) Active PMN unbound to spermatozoa, displaying early stages of extrusion of NET, characterised by filaments extending outward from the cell, derived from DNA from the cell’s nucleus. (C) Activated PMN phagocytosing ram spermatozoa via attachment at the head. (D) Activated PMN with fully extruded NET networks with two captured ram spermatozoa.

Air-dried slides were stained with a modified Wright’s stain according to the manufacturer’s instructions (1 min flood, wash twice with deionised water). Slides were examined using a phase-contrast microscope (Olympus CX41) at 200× magnification. A total of 200 PMNs were evaluated on each slide and classified as inactive (nucleus intact), active (disrupted nucleus/membrane) and free or bound to ≥1 spermatozoon. Images of interaction of PMNs with spermatozoa were captured using differential interference contrast (DIC) microscopy at 40× magnification on an Olympus BX53 microscope (Olympus, Australia; Fig. 1).

### C5 enzyme-linked immunosorbent assay (ELISA)

The concentration of complement C5 protein in the supernatant of the neutrophil binding assay and whole ram seminal plasma was assessed and quantified using a colourimetric human complement C5 ELISA kit (Abcam, AB125963, Australia) following the manufacturer’s instructions. Briefly, following 120 min incubation with PMNs, samples were stored at −80°. Samples were then thawed on ice and prepared by centrifugation at 1,500 ***g*** for 10 min at room temperature to remove any particulate matter. Samples and standards were assessed in duplicate in a pre-coated plate. A C5-specific biotinylated detection antibody was added followed by a washing procedure. Streptavidin-peroxidase conjugate was added and unbound conjugate was washed away. TMB was used to visualise the streptavidin-peroxidase enzymatic reaction, read on a TECAN Spark Multimode Microplate Reader (TECAN, Switzerland).

### Assessment of PMN viability

In study four, PMNs were isolated as described above and suspended in PBS at a concentration of 5 × 10^6^ PMN/mL. A 1:1 v/v dilution was performed with the PMN suspension and respective treatments (PBS, SSF, SP or S), resulting in a final concentration of 2.5 × 10^6^ PMN/mL, 25% v/v seminal plasma and 7.5% v/v serum. Samples were then incubated in a dry bath incubator (Major Science, USA) at 37°C. After 30 and 120 min, samples were mixed vigorously with a pipette to dissociate large cell clumps, and a 10 μL aliquot was incubated with 10 μL Trypan blue (5 min; room temperature), and cell viability was determined on a haemocytometer, counting 100 cells, as previously described (Pini *et al.* 2018*b*).

### Statistical analysis

All data were analysed using R (version 2023.03.0). Data were assessed for normality using the Shapiro–Wilk test and for homogeneity of variances using Levene’s test. Linear mixed models were fitted with sperm and serum treatments as fixed factors and ram and technical replicate as random factors. Pairwise comparisons were conducted and Tukey’s Honest Significant Difference (HSD) test was applied where appropriate to identify significant differences between treatments. Statistical significance was determined at *P* < 0.05. All values are reported as mean ± standard error of the mean.

## Results

### Visualisation of PMN binding response to ram spermatozoa

Following incubation of PMNs with spermatozoa, extrusion of NETs, degranulation and phagocytic activity were observed under DIC imaging (Fig. 1). Spermatozoa were entrapped in a network of convoluted fibres originating from activated PMNs (Fig. 1D). Spermatozoa were frequently captured either by their heads, midpieces or tails.

Furthermore, co-incubation with ram spermatozoa caused extensive PMN activation, with only a small proportion of PMNs observed with intact cell morphology (Fig. 1A). Activated cells exhibited rough or ruptured membrane surfaces and demarcation of the multi-lobular nucleus, resulting in darkening of the cytoplasm. There was clear activation of PMNs without direct sperm contact following 120 min of incubation (Fig. 1B), with cytoplasmic contents of the cell rupturing the membrane and extruding into the surrounding environment.

### PMN binding of both fresh and frozen sperm is increased in the presence of serum

The motility and percentage of viable, acrosome-intact fresh and frozen-thawed spermatozoa immediately before incubation with PMNs assessed in study 1 are reported below in Table 1.

**Table 1:** Total motility and viable, acrosome-intact spermatozoa (%) within fresh and frozen sperm treatments immediately before incubation with PMN. Data are presented as mean ± SEM across rams.

Study 1	Fresh	Frozen-thawed
Total motility (%)	63.59 ± 11.63	34.84 ± 10.27
Viability (%)	37.38 ± 6.87	14.38 ± 4.53

**Figure 2:**
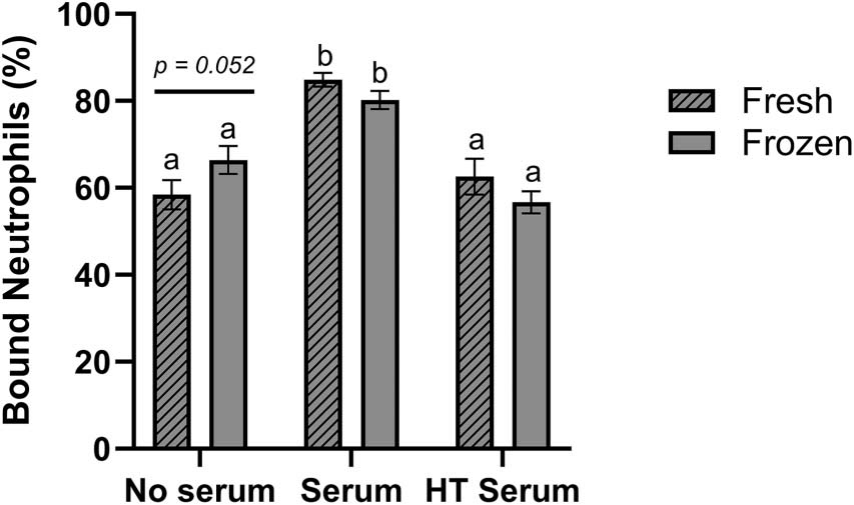
Percentage of neutrophils bound to ≥1 spermatozoa. Sperm incubated with isolated PMNs (no serum), 7.5% serum (serum), 7.5% heat-treated serum (HT serum). Data are presented as an average over ram (*n* = 3) and replicate (*n* = 3). Columns with different superscripts denote significant differences (*P* < 0.05) across treatments.

Quantification of sperm–neutrophil binding showed no significant difference in PMN binding after 30 min (Supplementary Table S2). After 120 min of co-incubation, a high rate of cellular binding to both fresh and frozen-thawed ram spermatozoa was recorded (68.6 ± 3%, 67.8 ± 2%, across all treatments; Fig. 2). The addition of serum significantly increased (*P* < 0.001) the rate of sperm–neutrophil binding in both fresh and frozen sperm treatments (84.8 ± 3%, 80.2 ± 3%) compared to no-serum treatments. When serum was heat-treated before incubation, binding returned to a similar level to serum-free conditions. Differences were observed in the binding of fresh and frozen-thawed sperm treatments in the serum-free treatments, approaching significance (*P* = 0.052).

### PMNs indiscriminate targeting of sperm viability, motility and diluent

Quantification of sperm–neutrophil binding showed no significant difference (*P* > 0.05) in PMN binding between ‘live’ (>75% viable, acrosome-intact) and ‘dead’ (0% viable, acrosome-intact) spermatozoa (75.8 ± 4%, 75.7 ± 5%, respectively). In addition, there was no significant difference (*P* > 0.05) in PMN binding between SSF and CRYO diluted spermatozoa (61.1 ± 12%, 58.4 ± 14%, respectively).

### Seminal plasma reduces neutrophil binding of frozen-thawed ram spermatozoa

The motility and percentage of viable, acrosome-intact frozen-thawed spermatozoa supplemented with SSF, SP or heat-treated SP (HTSP), assessed immediately before incubation with PMNs in study 3, are reported below in Table 2.

**Table 2:** Total motility and percentage of viable, acrosome-intact frozen-thawed spermatozoa supplemented with SSF, SP or HTSP, assessed immediately before incubation with PMN. Data are presented as mean ± SEM across rams.

Study 3	FT	FTSP	FTHTSP
Total motility (%)	31.13 ± 6.99	24.19 ± 7.36	20.91 ± 5.17
Viability (%)	20.05 ± 6.75	33.44 ± 13.50	33.44 ± 13.50

**Figure 3:**
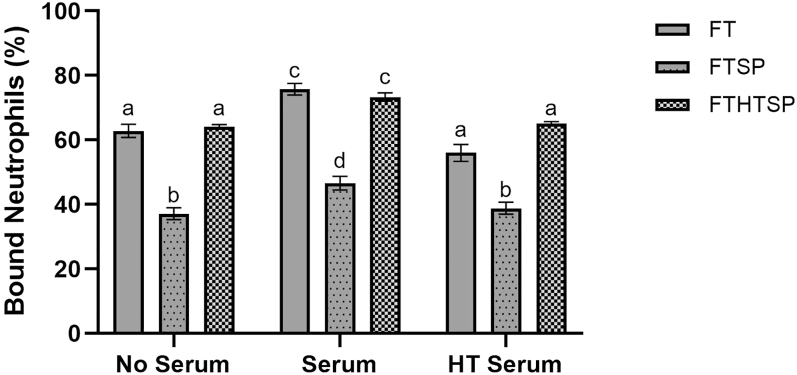
Percentage of neutrophils bound to ≥1 spermatozoa. Frozen-thawed spermatozoa (FT) and FT spermatozoa treated with 25% seminal plasma (FTSP) or heat-treated seminal plasma (FTHTSP) were incubated 1:1 (v/v) with isolated PMNs (no serum), 7.5% serum (S), 7.5% heat-treated serum (HTS). Data are presented as an average over ram (*n* = 3) and replicate (*n* = 4). Columns with different superscripts denote significant differences (*P* < 0.05) across treatments.

Quantification of sperm–neutrophil binding showed no significant difference in PMN binding after 30 min (Supplementary Table S3). After 120 min of co-incubation, frozen-thawed ram spermatozoa treated with 25% (v/v) seminal plasma (FTSP) showed a significant reduction (*P* < 0.001) in PMN binding across all serum treatments compared to the other treatments (FT and FTHTSP; Fig. 3). Serum had a significant effect on sperm–PMN binding (*P* < 0.001). Across all treatments, serum resulted in significantly higher binding levels compared to no or heat-treated serum, which were not significantly different from each other (*P* < 0.001, Fig. 3).

### Elevation of C5 levels in the presence of seminal plasma supplemented ram spermatozoa

**Figure 4:**
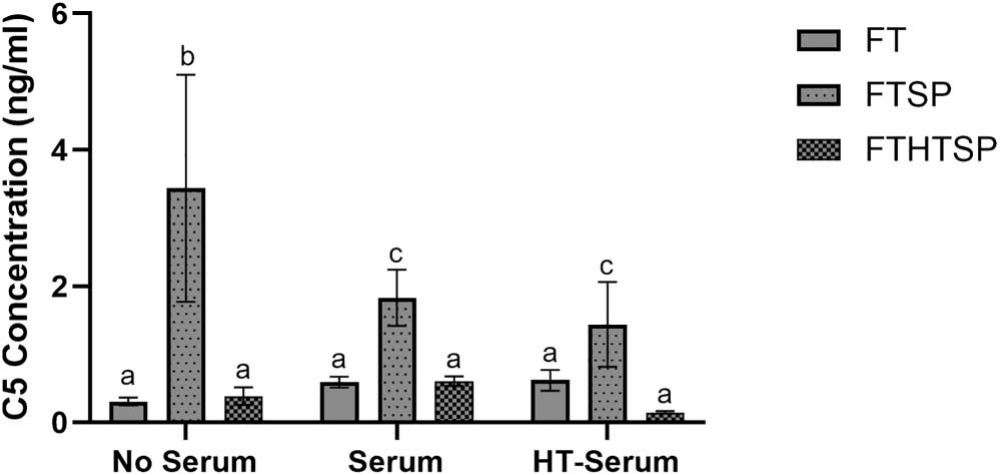
Levels of complement C5 protein in the supernatant of the neutrophil binding assay. Frozen-thawed sperm (FT), frozen-thawed sperm supplemented with 25% (v/v) seminal plasma (FTSP), and frozen-thawed sperm supplemented with 25% (v/v) heat-treated seminal plasma (FTHTSP) were incubated 1:1 (v/v) with isolated PMNs (no serum), 7.5% serum, 7.5% heat-treated serum (HT serum) for 120 min. Supernatant was then assessed for C5 concentration using ELISA. Data are presented as an average over pooled rams (*n* = 3) and replicates (*n* = 2).

Concentrations of C5 determined by ELISA showed that supplementation of ram spermatozoa with 25% (v/v) seminal plasma had a significant effect on supernatant C5 concentration (*P* < 0.05) across all serum treatments (Fig. 4). Regardless of serum treatment, FTSP had significantly higher levels of C5 compared to FT and FTHTSP. In the absence of serum, FTSP had a significantly higher C5 concentration (3.44 ± 1.6 ng/mL) than FT and FTHTSP (0.3 ± 0.05 ng/mL, 0.4 ± 0.12 ng/mL). The concentration of C5 in FTSP was similar in serum and heat-treated serum groups (1.83 ± 0.4 ng/mL, 1.44 ± 0.6 ng/mL), but significantly lower than in the no-serum group. There was no significant difference in C5 concentration in FT and FTHTSP sperm treatments across serum groups (*P* > 0.05). The overall intra-assay coefficient of variation (CV%) was 13.4%. The limit of detection (LOD) for the assay was calculated as 0.057 ng/mL.

Concentrations of C5 determined by ELISA showed that whole seminal plasma contains C5 in the absence of spermatozoa or serum. 25% seminal plasma contained 1.05 ± 0.01 ng/mL, while heat-treated seminal plasma contained 0.2 ± 0 ng/mL. C5 concentration decreased with concentration in 15% seminal plasma 0.29 ± 0 ng/mL but not 7.5% 0.25 ± 0 ng/mL.

### Viability of PMNs is not altered by serum or seminal plasma treatments

**Figure 5:**
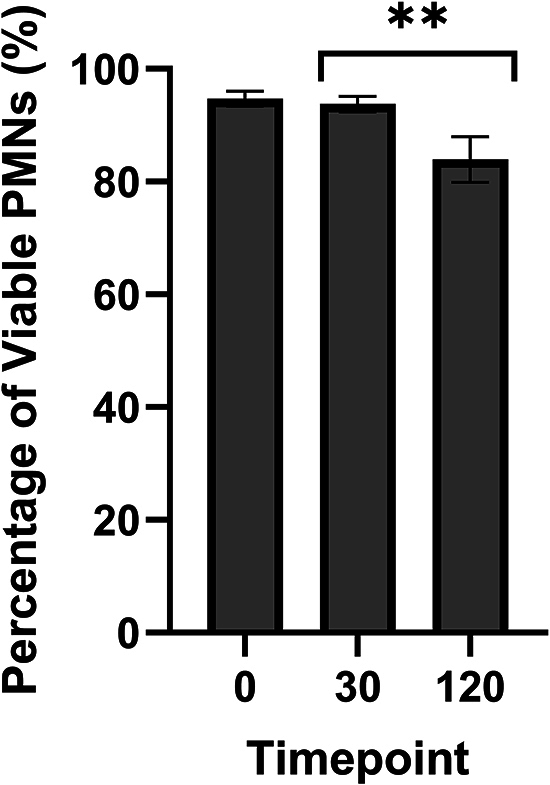
Viability of PMNs. Isolated PMNs were incubated with PBS, SSF, 25% v/v SP or serum, and viability was assessed via Trypan blue exclusion. Data are presented as an average over replicate (*n* = 4) and treatment. Columns with asterisks denote significant differences (*P* < 0.01) across time points.

There was no interaction of treatment and time point on the viability of PMNs assessed by Trypan blue staining, following incubation with PBS, SSF, 25% SP or 7.5% serum. When pooled over time points, there was also no effect of treatment (*P* > 0.05; Supplementary Table S4). When pooled over treatment, the viability of PMNs declined significantly at 120 min (*P* < 0.01; Fig. 5).

## Discussion

The present study presents new evidence for the immunosuppressive properties of seminal plasma in sheep which could be leveraged to aid transit through the cervix. Results validate the use of neutrophil binding assays as a valuable *in vitro* tool for studying immune dynamics between sperm and female-derived cells. Despite not reaching significance, there was a clear trend towards frozen-thawed sperm being more susceptible to neutrophil binding compared to fresh sperm in the absence of serum. This was not attributed to the viability of spermatozoa post-thaw or the presence of specific diluent components such as egg yolk. In the presence of whole or heat-treated serum, this apparent differential targeting of PMNs to fresh and frozen spermatozoa was not observed. Irrespective of sperm phenotype, serum significantly increased sperm–neutrophil binding. However, this was reduced to serum-free levels following heat treatment, suggesting sperm–neutrophil interaction dynamics are further mediated by protein immune components within ewe serum which likely mirrors fluids within the female reproductive tract (Chemonges *et al.* 2016, Soleilhavoup *et al.* 2016). The supplementation of seminal plasma to frozen-thawed spermatozoa significantly reduced sperm–PMN binding across all serum groups. This protective effect was lost, however, when seminal plasma was heat-treated before incubation, suggesting the factor protecting spermatozoa from immune attack is heat-labile. Interestingly, complement C5 levels in the supernatant collected post-incubation showed the inverse relationship to neutrophil binding, with treatments incubated with seminal plasma containing higher levels of C5 compared to sperm-only treatments. Assessment of raw seminal plasma from the same rams confirmed this result, with high levels of C5 detected. Combined, these results suggest that C5 in seminal plasma could modulate the immune response to frozen sperm via action on terminal products of the complement cascade. Further investigation into the anti-complement activity of ram seminal plasma is required to better understand its mechanistic interaction with the female immune response. This relationship could be an important target to improve the passage of frozen-thawed sperm through the ovine cervix.

Previous studies in species such as donkeys have attempted to show differences between the susceptibility of fresh and frozen-thawed ram spermatozoa to neutrophils (Miró *et al.* 2013). In the current study, frozen-thawed ram spermatozoa showed increased susceptibility to neutrophil binding compared to fresh spermatozoa in a serum-free environment; however, this difference did not reach significance. Differences between fresh and frozen ram sperm also failed to reach significance in a previous study by Pini *et al.* (2018*b*). However, the study noted a significant effect of diluent-derived egg yolk on sperm–PMN interactions, suggesting this may cause a failure in opsonin-mediated binding by neutrophils. Conversely, a study using equine spermatozoa showed no effect of egg yolk on sperm–neutrophil binding (Alghamdi *et al.* 2009). To address this, the protocol was modified to wash all sperm treatments of residual diluent before neutrophil incubation, reducing any diluent effects compounding results. In the current study, neither sperm viability nor prior exposure to egg yolk was shown to influence sperm–neutrophil binding, as there was no significant difference between egg-yolk-free (SSF) and egg yolk (CRYO) diluted spermatozoa, suggesting that these differences in treatment are unlikely to be responsible for the difference observed in treatments above.

Fresh and frozen-thawed ram spermatozoa differ in their molecular profile, with cryopreservation altering the abundance of 51 proteins (Pini *et al.* 2016, 2018*a*). While not yet tested, several of these protein alterations in frozen sperm could influence immune recognition and susceptibility. Notably, of these 51 proteins, cryopreservation resulted in an increased abundance of phospholipid scramblase 1 (PLSCR1), which is known to externalise phosphatidylserine residues and promote the recognition and clearance of immune cells including neutrophils (for review see Dal Col *et al.* (2022)). In addition, frozen sperm showed reduced expression of protective proteins such as heat shock proteins (HSP70 and HSP75) and disintegrin/metalloproteinase domain-containing protein 20-like (ADAM20-like), both of which have roles in maintaining membrane integrity and modulating immune interactions including cell signalling, adaptive and innate immune activation, immune privilege and inflammation (for review see Klein & Bischoff (2011), Lambrecht *et al.* (2018), Zininga *et al.* (2018), Hu *et al.* (2022)). The upregulation of leucocyte elastase inhibitor in frozen sperm also could suggest an attempt to counteract immune-mediated degradation, albeit potentially insufficient to prevent heightened neutrophil engagement. Together with the current findings, molecular alterations during cryopreservation support the hypothesis that frozen sperm may be more immunogenic than fresh sperm, being more susceptible to the immune response, consistent with their observed impaired transit through the reproductive tract. While the results highlighted a trend indicating differences between fresh and frozen sperm in the no-serum group, the neutrophil binding assay used in the current study may not be sensitive enough to replicate these differences occurring *in vivo*. We suggest that the inclusion of additional measures of immune cell interaction and cell signalling mediators be considered in future studies in an effort to further elucidate molecular differences in immune susceptibility of frozen spermatozoa.

To further investigate the mechanisms of sperm–neutrophil binding, assays were performed in the presence and absence of ewe serum. This approach enabled the assessment of non-opsonin-mediated binding (e.g. via selectins, lectins and integrins) in a serum-free environment as well as opsonin-mediated binding (e.g. via immunoglobulins and C-reactive protein) in the presence of serum, with complement inactivation achieved through heat treatment. In the presence of serum, we saw significant amplification of neutrophil binding. The addition of serum increases the availability of precursory factors for the complement cascade (Sarma & Ward 2011). At least within our experimental assay, complement-mediated responses, namely opsonisation, play a major role in sperm–neutrophil interactions. The initiation of the alternate complement pathway is dependent on the availability of complement C3 protein, which is hydrolysed to form C3a and C3b. C3b binds the Bb molecule derived from factor B to form C3bBb which is bound to the cell surface (Sarma & Ward 2011). Human serum has been shown to contain both C3 and factor D in its active form. Factor D is thus continually available for the C3 convertase complex (C3bBb) via activation and cleavage of factor B (Volanakis *et al.* 1993). Consequently, when in the presence of serum, neutrophils have all constituents to activate the alternate pathway, leading to the generation of terminal complement products such as C3b (opsonisation), C3a/C5a (inflammation) and C5b-9 (MAC) (Sarma & Ward 2011). The presence of these factors amplifies the feedback loop within the complement cascade, resulting in an increased inflammatory response and subsequent release of mediators which increase the leukocytic activity of PMNs. The amplification of the alternate pathway in the presence of serum supports the observed increase in PMN interactions with sperm in the present study. Furthermore, heat treatment of serum universally led to lower binding of sperm, irrespective of sperm type. Both factor D and C3 are proteins (Sarma & Ward 2011), and as such, heat treatment of serum may have resulted in their denaturation, diminishing the alternate pathway amplification of neutrophil opsonisation. These findings further support that complement may be a major sperm–neutrophil binding mechanism (Clark & Klebanoff 1976, Troedsson *et al.* 1998, Rozeboom *et al.* 2001).

The inclusion of seminal plasma with incubated PMNs revealed a critical role of seminal plasma in significantly reducing PMN binding of frozen-thawed sperm. This is likely a physiological protective mechanism which may serve to suppress excessive immune responses in the female tract and allow for sperm transport (Schjenken & Robertson 2020). In the present study, SP was added to washed spermatozoa at 50% v/v, which is within the realms of what is considered physiologically relevant (in terms of the spermatozoa: SP ratio (Mann 1964)) and what has been considered in previous studies by Alghamdi *et al.* (2009), who tested the influence of 0, 10, 20 and 40% SP on PMNs. The final concentration of 25% v/v SP following 1:1 dilution with suspended PMNs reflects a careful balance of what is physiologically relevant and logistically possible within the constraints of the experiment; however, future studies measuring a dose-dependent response of seminal plasma on sperm–neutrophil interactions would be interesting. Nevertheless, the reported effect of seminal plasma was observed across all serum treatments, suggesting its inhibitory mechanisms may operate in the shared later stages of the cascade, irrespective of activation pathway. The protective effect of seminal plasma on neutrophil interactions with spermatozoa reported in the present study has previously been reported in humans, horses, donkeys, cattle and sheep (D’Cruz & Haas 1995, Alghamdi *et al.* 2004, Oren-Benaroya *et al.* 2007, Miró *et al.* 2013, Pini *et al.* 2016). However, studies in cattle have described elevated leucocyte binding and phagocytosis in the presence of seminal plasma (Alghamdi *et al.* 2009). These differences may be attributed to differences in the location of semen deposition (vagina vs uterus) or collection methodology for seminal plasma (artificial vagina vs electroejaculation), which may impact its molecular composition (Mattner & Voglmayr 1962). The protective effect of seminal plasma in the stallion has previously been attributed to CRISP3 (Doty *et al.* 2011) and DNases (Alghamdi & Foster 2005); however, to date, the responsible factors have yet to be confirmed. Reproductive immunology studies in sheep are very limited despite significant incentive derived from their unique fertility limitation within the cervix (Warr *et al.* 2023). From the current study, we hypothesise that mediating factors of sperm–PMN interactions could likely be seminal plasma proteins, given that their protective effect was lost following heat treatment, and thereby are heat-labile in nature.

We propose that a major element of seminal plasma responsible for reduced sperm–neutrophil binding may be proteins involved in the complement cascade, particularly C5. Analysis of C5 levels in the supernatant collected post sperm–PMN incubation showed an inverse relationship to neutrophil binding, with seminal plasma treatments containing higher levels compared to sperm-only treatments. In addition, C5 abundance was also reduced following seminal plasma heat treatment. The discovery that whole ram seminal plasma contains C5 was also interesting and, to the best of our knowledge, has not been previously described (Soleilhavoup *et al.* 2014, Rickard *et al.* 2015, Leahy *et al.* 2019). C5 is common across all complement pathways. It has previously been suggested as a useful agent to quantify levels of complement activity in seminal plasma (Taylor *et al.* 2008, Alghamdi *et al.* 2009), given it is consequently cleaved by C5 convertase, initiating activation of the terminal complement membrane attack complex (MAC) (Sarma & Ward 2011). C4 and C3 have previously been reported in human SP, with ‘little evidence of functionality’ (Sullivan & Quinlivan 1974, Blenk & Hofstetter 1991, Rahimi *et al.* 1999, Boit *et al.* 2003). Functionality of C5 is dependent on its activation, being cleaved into C5a and C5b (Vogt *et al.* 1978). The cleavage is dependent on C3b which has been fixed to a cellular surface immediately after its release from C3 by a surface-fixed enzyme (Vogt *et al.* 1978). Efficient cleavage is possible only when C3b is free of other ligands such as factor B or properdin (Vogt *et al.* 1978). Accumulated C5 in the supernatant of our seminal plasma-treated samples may suggest reduced cleavage of C5 into its active products C5a and C5b, which are involved in the recruitment of inflammatory cells and the formation of MAC responsible for cell lysis (Nicholson-Weller & Halperin 1993, Guo & Ward 2005). Human seminal plasma contains membrane cofactor protein (CD46) and clusterin (CLU), which are known inhibitors of MAC and regulators of complement activation (Vanderpuye *et al.* 1992). CD46 inactivates C3 and C5 convertases, which are responsible for the cleavage of CD46 and CLU into their terminal products. Interestingly, CLU has previously been identified in ram seminal plasma (Rickard *et al.* 2015). Human CLU has been shown to inhibit C5b-9-initiated complement cell lysis (Vanderpuye *et al.* 1992). CD46 and CLU are just two of many fluid-phase and membrane-bound complement regulatory (CReg) proteins that tightly control this system, avoiding tissue damage to the host. The presence of C5 in ram seminal plasma is an interesting discovery that furthers our hypothesis that complement-mediated immune responses are pivotal in sperm–neutrophil dynamics within the female reproductive tract. It would be interesting to study whether clusterin or other CReg proteins identified in ram seminal plasma reduce sperm–neutrophil binding through inhibition of the complement pathway in future studies.

It is increasingly evident that the complex dynamics between seminal fluid and the female immune system contribute to the ability of a spermatozoon to ascend the female reproductive tract. It remains unclear whether changes incurred during *in vitro* processing and cryopreservation may interfere with this sensitive dynamic, increasing immune attack and clearance. Regardless, seminal plasma appears to protect frozen-thawed spermatozoa against neutrophil binding via an unidentified heat-labile factor *in vitro*. Mediation of sperm–PMN interactions via the alternate complement pathway could explain the effect of serum and even seminal plasma in the present study; however, to date, this has not been experimentally tested and should be addressed in future studies. Notably, we also presented the novel finding of complement factor C5 in ram seminal plasma. The exact mechanisms underpinning the observed sperm–PMN interactions remain undefined, highlighting the substantial gaps in our fundamental understanding of sperm dynamics with the female immune system in sheep. Ovine studies in this area remain sparse, and future studies focussing on the mechanisms underpinning the observed sperm–PMN interactions and the modulatory factors within seminal plasma would greatly accelerate investigations into the complex requirements necessary for successful cervical transit.

## Supplementary materials



## Declaration of interest

The authors declare that there is no conflict of interest that could be perceived as prejudicing the impartiality of the work reported.

## Funding

This work was supported by funding from the Late Dorothy Minchin Bequest, generously provided by the Sydney School of Veterinary Science, The University of Sydney. S Warr was supported by the Thomas Lawrence Pawlett Scholarship.

## Author contribution statement

Conceptualization was carried out by SW and JPR. Resources were provided by JPR. Original draft preparation was completed by SW. Writing, review and editing was performed by SW, TP, SPdG and JPR. Supervision was provided by JPR. Project administration was undertaken by JPR.
